# The independent association of myocardial extracellular volume and myocardial blood flow with cardiac diastolic function in patients with type 2 diabetes: a prospective cross-sectional cohort study

**DOI:** 10.1186/s12933-023-01804-9

**Published:** 2023-03-31

**Authors:** Annemie S. Bojer, Martin H. Sørensen, Stine H. Madsen, David A. Broadbent, Sven Plein, Peter Gæde, Per L. Madsen

**Affiliations:** 1 Department of Cardiology and Endocrinology, Slagelse Hospital, Ingemannsvej 32, Region Zealand, 4200 Slagelse, Denmark; 2 Institute of Regional Health Research, Faculty of Health Sciences, University of Southern, Odense, Denmark; 3 Department of Cardiology, Copenhagen University Hospital Herlev-Gentofte, Capital Region of Denmark, Hellerup, Denmark; 4 Department of Medical Physics and Engineering, Leeds Teaching Hospitals NHS Trust, Leeds, UK; 5 Leeds Institute of Cardiovascular and Metabolic Medicine, University of Leeds, Leeds, UK; 6 Department of Clinical Medicine, University of Copenhagen, Copenhagen, Denmark

**Keywords:** Diabetes, Diabetes complications, Cardiac diastolic function, Cardiac magnetic resonance imaging, Myocardial extracellular volume, Myocardial perfusion reserve, Myocardial interstitial fibrosis, Myocardial microvascular function

## Abstract

**Background:**

Diffuse myocardial fibrosis and microvascular dysfunction are suggested to underlie cardiac dysfunction in patients with type 2 diabetes, but studies investigating their relative impact are lacking. We aimed to study imaging biomarkers of these and hypothesized that fibrosis and microvascular dysfunction would affect different phases of left ventricular (LV) diastole.

**Methods:**

In this cross-sectional study myocardial blood flow (MBF) at rest and adenosine-stress and perfusion reserve (MPR), as well as extracellular volume fraction (ECV), were determined with cardiovascular magnetic resonance (CMR) imaging in 205 patients with type 2 diabetes and 25 controls. Diastolic parameters included echocardiography-determined lateral e’ and average E/e’, and CMR-determined (rest and chronotropic-stress) LV early peak filling rate (ePFR), LV peak diastolic strain rate (PDSR), and left atrial (LA) volume changes.

**Results:**

In multivariable analysis adjusted for possible confounders including each other (ECV for blood flow and vice versa), a 10% increase of ECV was independently associated with ePFR/EDV (rest: β = − 4.0%, stress: β = − 7.9%), LA_max_ /BSA (rest: β = 4.8%, stress: β = 5.8%), and circumferential (β = − 4.1%) and radial PDSR (β = 0.07%/sec). A 10% stress MBF increase was associated with lateral e′ (β = 1.4%) and average E/e’ (β = − 1.4%) and a 10% MPR increase to lateral e′ (β = 2.7%), and average E/e’ (β = − 2.8%). For all the above, p < 0.05. No associations were found with longitudinal PDSR or left atrial total emptying fraction.

**Conclusion:**

In patients with type 2 diabetes, imaging biomarkers of microvascular dysfunction and diffuse fibrosis impacts diastolic dysfunction independently of each other. Microvascular dysfunction primarily affects early left ventricular relaxation. Diffuse fibrosis primarily affects diastasis.

*Trial registration* https://www.clinicaltrials.gov. Unique identifier: NCT02684331. Date of registration: February 18, 2016.

**Supplementary Information:**

The online version contains supplementary material available at 10.1186/s12933-023-01804-9.

## Background

The pathophysiology of a failing heart in patients with type 2 diabetes is multifactorial and still not fully understood [1]. Diastolic dysfunction is highly prevalent [1, 2] and has been linked with poor outcomes [3]. The most prevalent type of heart failure in patients with type 2 diabetes is heart failure with preserved ejection fraction (HFpEF) [4]. Identifying those patients with type 2 diabetes with cardiac involvement is becoming increasingly important in light of sodium-glucose cotransporter 2 inhibitors (SGLT-2i) treatment proven to reduce hospitalization for heart failure [5]. Impaired microvascular function from microangiopathy and the development of interstitial diffuse fibrosis are generally believed to be parts of the pathogenesis leading to diastolic dysfunction [1]. The evidence for this connection stems from animal studies [6] and smaller human biopsy studies [7]. Non-invasively the myocardial extracellular volume (ECV), a biomarker of fibrosis, and myocardial blood flow (MBF) can be quantified with cardiovascular magnetic resonance (CMR) imaging [8, 9] within larger human patient populations. MBF [10] and ECV [11] have sporadically been related to impaired LV filling, but their relative importance is unknown since they have not been systematically studied within the same cohort. Prior studies have demonstrated a correlation between ECV and MBF at stress [12]. Theoretically, increased ECV caused by interstitial fibrosis could lead to impaired microvascular function or vice versa; thus, whether they are important independently from each other is unclear. The very early diastole relates to cardiomyocyte relaxation which is an energy-requiring process**,** whereafter LV compliance (myocardial stiffness) becomes increasingly important [13].

The objective of this study was to assess the association of ECV and MBF (at rest and adenosine stress) with clinically relevant parameters of LV filling reflecting both myocardial relaxation and LV compliance in a cross-sectional study of patients with type 2 diabetes. We hypothesized that both ECV and MBF would be associated with LV diastolic function but that they would be so independently of each other. We hypothesized ECV and MBF to affect different phases of diastole in patients with type 2 diabetes. ECV, a biomarker of fibrosis, would be likely to affect LV compliance; on the other hand, MBF is directly related to oxygen delivery to the myocardium and hence is hypothesized to be associated with the very early energy-requiring relaxation of the cardiomyocytes.

## Methods

### Study design and population

The study protocol has been reported previously [12, 14, 15]. In short, this was a cross-sectional study of 296 patients with type 2 diabetes recruited from the outpatient clinic at the Endocrinology Department at Naestved-Slagelse-Ringsted (NSR) Hospital in Denmark. Further, 25 age- and sex-matched control subjects were included in whom statin therapy for hypercholesterolemia and well-controlled hypertension requiring only one drug treatment was allowed. The study was approved by the local ethics committee of region Zealand (SJ-490) and by the Danish Data Protection Agency (REG-167-2015) and complied with the Declaration of Helsinki. The study was registered at www.clinicaltrials.gov with the unique identifier NCT02684331 and the STROBE recommendation for reporting cross-sectional studies was followed. From February 2016 until July 2019, patients were enrolled after written informed consent. We included patients with type 2 diabetes between 18 and 80 years of age. We excluded patients with claustrophobia, permanent or persistent atrial fibrillation, or an estimated glomerular filtration rate (eGFR) < 30 mL/min/1.73 m^2^ (a contraindication for gadolinium contrast). If the patients had a contraindication to glycopyrrolate, they were included, but the CMR protocol was performed without this part. For this particular study, we additionally excluded patients with prior coronary artery bypass surgery; because their myocardial circulation is altered, our tool for quantifying myocardial blood flow could not be applied. Information on prior medical history, current medication, and a physical examination, including assessment of retinopathy, nephropathy, and neuropathy, was obtained, and echocardiography and CMR imaging were performed within 14 days. Further, urine and blood sampling were obtained as previously described [12, 14, 15]. As a measure of the oxygen demand of the myocardium, the rate pressure product (RPP) was calculated as RPP = heart rate (beat per minute) _*_ systolic blood pressure (mmHg) [16].

### Echocardiography

An echocardiogram was obtained with a General Electric (GE) Healthcare (Illinois, USA) Vivid E9 ultrasound system. For this study, only parameters for diastolic function were used. With echo-Doppler, the peak early mitral inflow (E), the diastolic early myocardial tissue velocity (e′) was measured, and the E/e′ ratio was calculated, as previously described [14]. The analysis was performed immediately after imaging without blinding. However, the analysis was performed before knowledge of ECV or MBF/MPR was available.

### Cardiovascular magnetic resonance

**Fig. 1:**
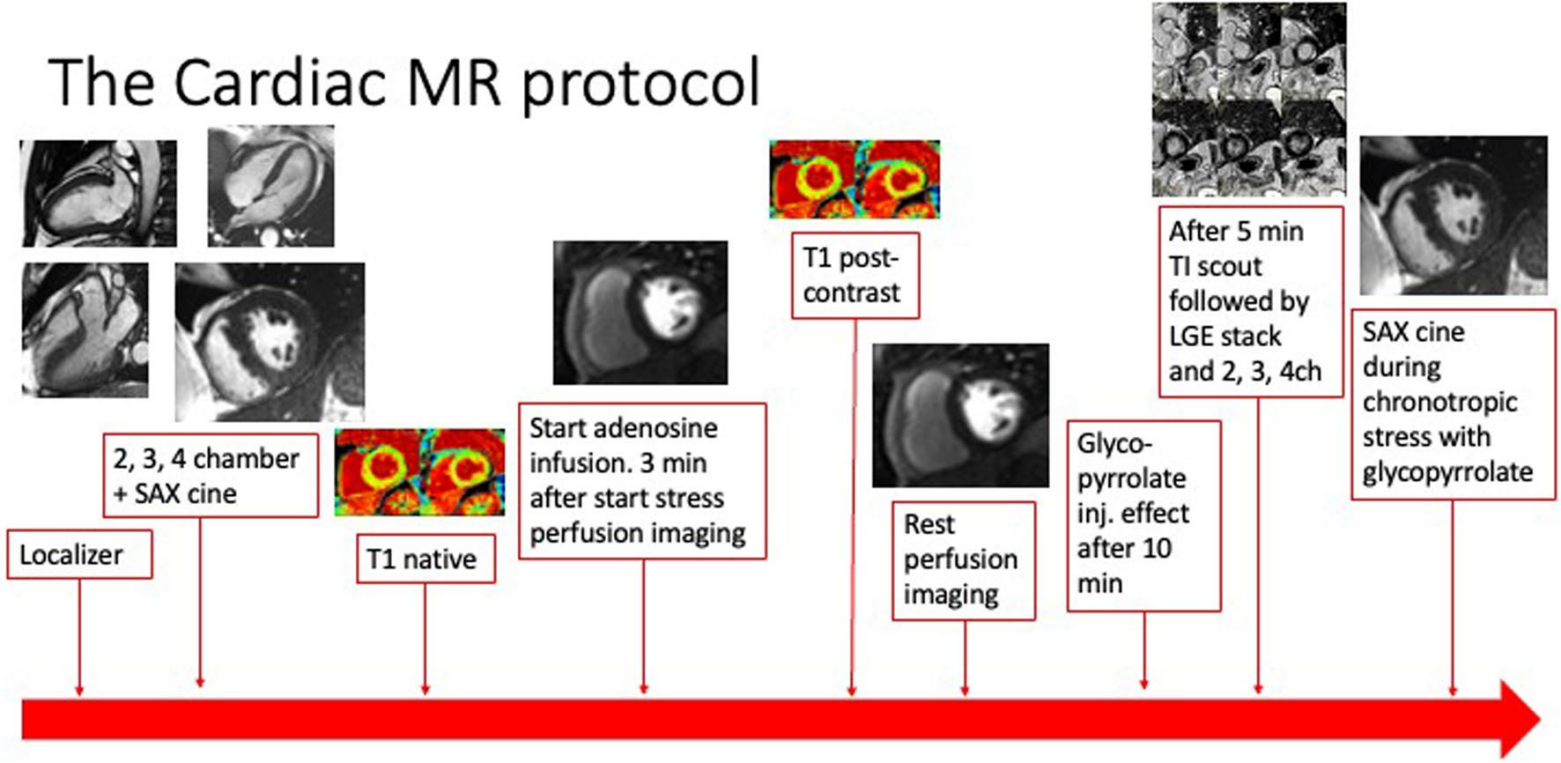
The cardiovascular magnetic resonance protocol

Patients were scanned on a 1.5 T Siemens Avanto (Siemens Healthineers, Erlangen, Germany). The CMR protocol has been described previously [12, 14, 15]. A graphic overview of the protocol is presented in Fig. 1. Surface and spine coils were used with patients in a supine position. Following scout images, cardiac 2-, 3-, and 4-chamber cine images and short-axis steady-state free precession cine images were obtained. Images were acquired during end-expiratory breath-holds (25 phases; slice thickness 8 mm, no gap; TE 1.16—1.25 ms; TR 46.24—49.98 ms, matrix 210–208; FoV 258 × 320–485 × 481). Short-axis images were repeated 10 min after an intravenous bolus injection of the chronotropic stressor glycopyrrolate (4 μg/kg; Robinul^®^, Mylan, Denmark), which has previously been shown to accentuate diastolic dysfunction [17]. In post-processing analysis (cvi42, Circle Cardiovascular Imaging, Calgary, Canada, v.5.13.5), left atrial (LA) and LV time-volume curves were generated from the shot-axis cine images by semi-automatic tracing of the endocardial borders in all 25 phases. Maximal (LA_max_), minimal (LA_min_), and mid-diastolic LA volumes (LA_mdv_) were determined. LA_max_ was indexed to body surface area (BSA) (Mosteller). The LA total emptying fraction (LAEF) was calculated as (LA_max_ – LA_min_)/LA_max_ * 100%, and the LA passive emptying fraction (LA_PEF_) was determined as (LA_max_ – LA_mdv_)/LA_max_ * 100%. On LV time-volume curves, the early peak filling rate (ePFR) was generated automatically. The ePFR was indexed to LV end-diastolic volume (EDV).

T1 mapping was obtained at a basal and mid-ventricular short-axis slice using a shortened modified Look-Locker inversion recovery sequence [18]. Native T1 maps (non-contrast) were acquired before stress perfusion, and T1 post-contrast maps were acquired 10 min after. The extracellular volume fraction (ECV) was estimated as:

1 $$ECV = \left( {1 - haematocrit} \right)\left( {\frac{{{\raise0.7ex\hbox{$1$} \!\mathord{\left/ {\vphantom {1 {T1_{post - contrast.myocardium} - }}}\right.\kern-0pt} \!\lower0.7ex\hbox{${T1_{post - contrast.myocardium} - }$}}{\raise0.7ex\hbox{$1$} \!\mathord{\left/ {\vphantom {1 {T1_{native.myocardium} }}}\right.\kern-0pt} \!\lower0.7ex\hbox{${T1_{native.myocardium} }$}}}}{{{\raise0.7ex\hbox{$1$} \!\mathord{\left/ {\vphantom {1 {T1_{post - contrast.blood} - }}}\right.\kern-0pt} \!\lower0.7ex\hbox{${T1_{post - contrast.blood} - }$}}{\raise0.7ex\hbox{$1$} \!\mathord{\left/ {\vphantom {1 {T1_{native.blood} }}}\right.\kern-0pt} \!\lower0.7ex\hbox{${T1_{native.blood} }$}}}}} \right)$$

$E$Average ECV was calculated from the basal and mid-ventricular slices [18]. As per guidelines, areas with ischemic late gadolinium enhancement (subendocardial) were excluded, but areas with non-ischemic late gadolinium enhancement were included [18]. As previously reported 28 subjects had non-ischemic late gadolinium enhancement lesions [14]. The pattern was distinct for this cohort and did not resemble the pattern of myocarditis or other cardiomyopathies. Without reference, the 2017 SCMRI guidelines suggest performing T1 post-contrast mapping after a gadolinium dose of 0.1–0.2 mmol/kg, whereas we performed T1 post-contrast mapping after a dose of 0.075 mmol/kg as recommended in Denmark and in some other CMR sites, [19, 20]. ECV quantification has not been shown to be significantly gadolinium dose-dependent, and in fact highly robust to gadolinium dose, scanner strength and time after gadolinium administration. The ECV values of our control patients was equal to normal age- and sex-matched normal values with a dose of 0.1–0.2 mmol/kg. However, even with such robust scans to increase the for external validation, importantly, ECV is age- and sex-dependent [21] and an age- and sex-matched control group must still be recommended and was consequently included in this study.”

MBF was assessed on a mid-ventricular short-axis slice at rest and during adenosine stress (140 µg/kg/min) using gadolinium contrast (0.075 mmol/kg Gadovist; Bayer AG) as previously described [12]. Patients with previous CABG surgery were excluded as mentioned above. During the quantification of MBF, we carefully excluded areas of reversible or irreversible ischemic perfusion defects on the perfusion images as well as areas with ischemic late gadolinium enhancement (subendocardial).MBF was quantified at rest and during stress using in-house MATLAB 2015b (MathWorks, Natick, MA) code. Myocardial perfusion reserve (MPR) was calculated as the ratio of stress MBF to rest MBF.

Myocardial peak diastolic strain rates were measured with 2D feature tracking on CMR 2-, 3-, and 4-chamber cine images (longitudinal) and the short axis cine stack (circumferential and radial) at rest.

### Diastolic parameters

**Fig. 2:**
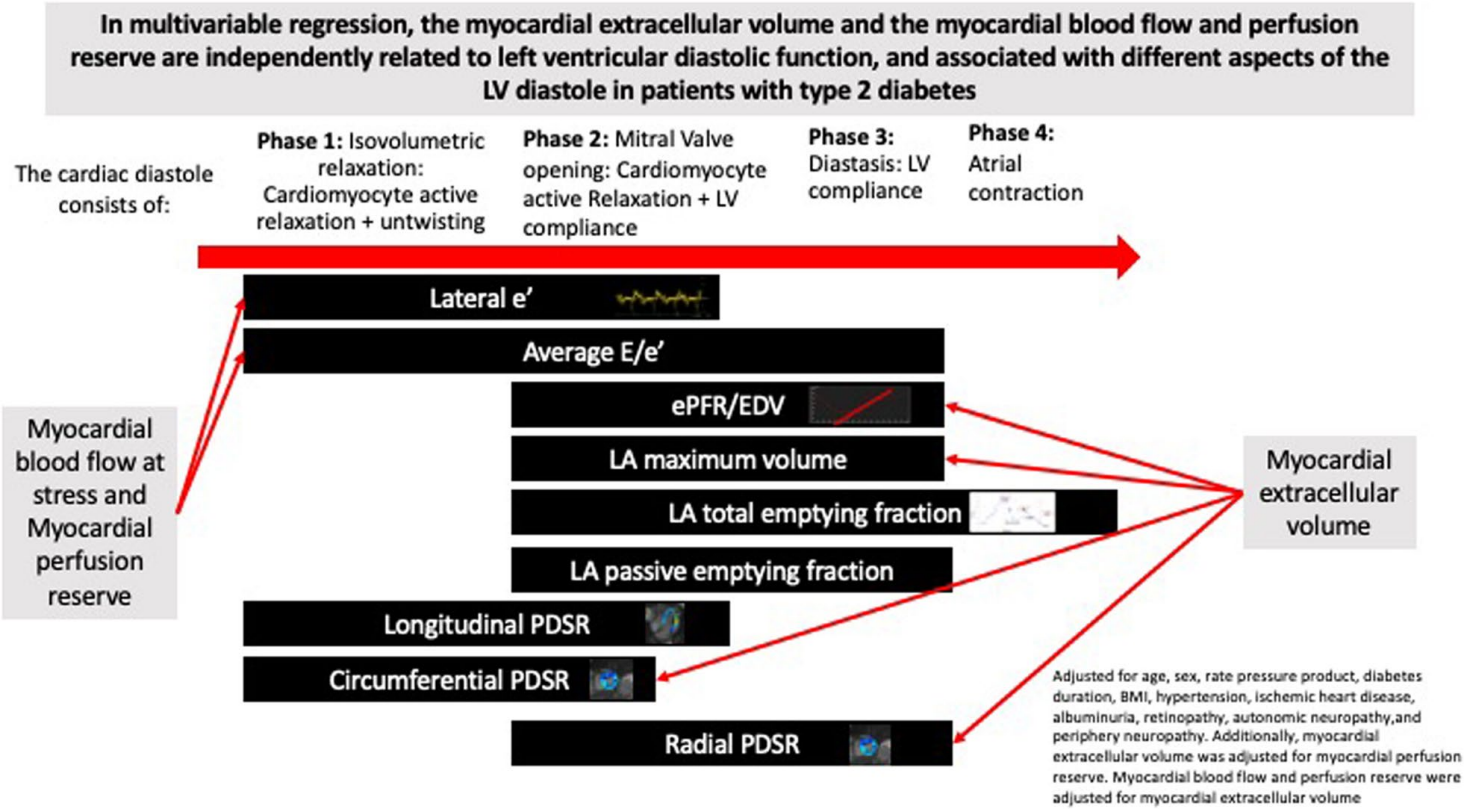
The relationship between the phases of the left ventricular diastole and the imaging parameters that were measured with echocardiography and cardiac magnetic resonance imaging

The left ventricular diastole can be divided into four phases (Fig. 2) [13]. The different phases of the LV diastole and the different underlying mechanisms are, albeit with some overlap, evaluated from different diastolic echocardiographic and CMR parameters [13, 22–24]. For this study, we choose the two most used echocardiographic parameters, namely the lateral e´ and the average E/e´. They reflect important parts of the early LV diastole and do not suffer from pseudo-normalization throughout the stages of diastolic dysfunction [13]. Additionally, the latter has been found to be of clinical importance in patients with type 2 diabetes [25]. We choose ePFR/EDV because this parameter has been shown to identify patients with HFpEF [26] in previous work, and the LA volume and volume changes during diastole (LA_max_, LA_PEF_, LAEF) which have been linked to poor outcomes [26]. Additionally, we assessed ePFR/EDV and LA volume changes during chronotropic stress, which has previously been shown to reveal masked diastolic dysfunction [17]. Lastly, the newer peak diastolic strain rate was chosen both because they are increasingly used in the clinic and because they have been shown to detect small, subtle myocardial dysfunction [26]. In short, we aimed at a plethora of parameters enabling us to reflect all parts of the diastole reflecting both myocardial relaxation and the LV wall compliance, as both must be considered of importance for LV filling.

Analysis of echo-Doppler and CMR variables diastolic variables were performed without blinding of the clinical patients information, but before knowledge of ECV or MBF/MPR was available.

### Statistical analysis

Continuous variables are presented as mean and standard deviation or median and interquartile range (IQR) as appropriate and were compared using an unpaired two-tailed Student t-test or Mann–Whitney U test. Categoric variables were presented as counts and percentages and compared using a chi-squared test or Fisher’s exact test as appropriate.

Associations between ECV, MBF (rest, stress, and the MPR, respectively), and LV filling parameters were assessed in patients with type 2 diabetes in two general multivariable linear regression models, control subjects were excluded from these analyses. In a basic model, adjustments were made for age (≤ 50, 51–64, ≥ 65 years of age), sex, and RPP, all three with previous well-established effects on LV filling, ECV, and MBF [13, 27]. Due to collinearity with RPP, heart rate was deselected from the model. In a large model, we additionally included the duration of diabetes (≤ 10, 11–19, ≥ 20 years), body mass index (BMI; ≤ 25.0, 25.1–29.9, ≥ 30 kg/m^2^), hypertension, ischemic heart disease, albuminuria, retinopathy, autonomic neuropathy, and periphery neuropathy, in addition to ECV and MBF (rest, stress or MPR respectively). Thus, in the large multivariable model, associations with ECV were adjusted for perfusion indices and vice versa. LV filling, ECV, and MBF are related to age and sex; therefore, we chose not to report a univariable model [13, 18, 22]. Parameters in the multivariable model were all factors suspected to be associated with LV filling and/or worsening of diabetic heart disease. We chose to include the parameters based on directed acyclic graphs because this is considered superior to older methods for parameter selection because all possible confounders are included in the model [28]. Assumptions for the general linear model were checked, and the outcome variable was transformed as appropriate. The beta values were transformed to represent an increase of 10% in ECV, MBF, and MPR. Sensitivity analyses were performed to assess differences between patients included in this study and the patients in the total cohort but excluded from this study. A two-sided p < 0.05 was considered statistically significant. Statistical analysis was performed with R studio version 1.2.1093 (R Development Core Team).

## Results

**Fig. 3:**
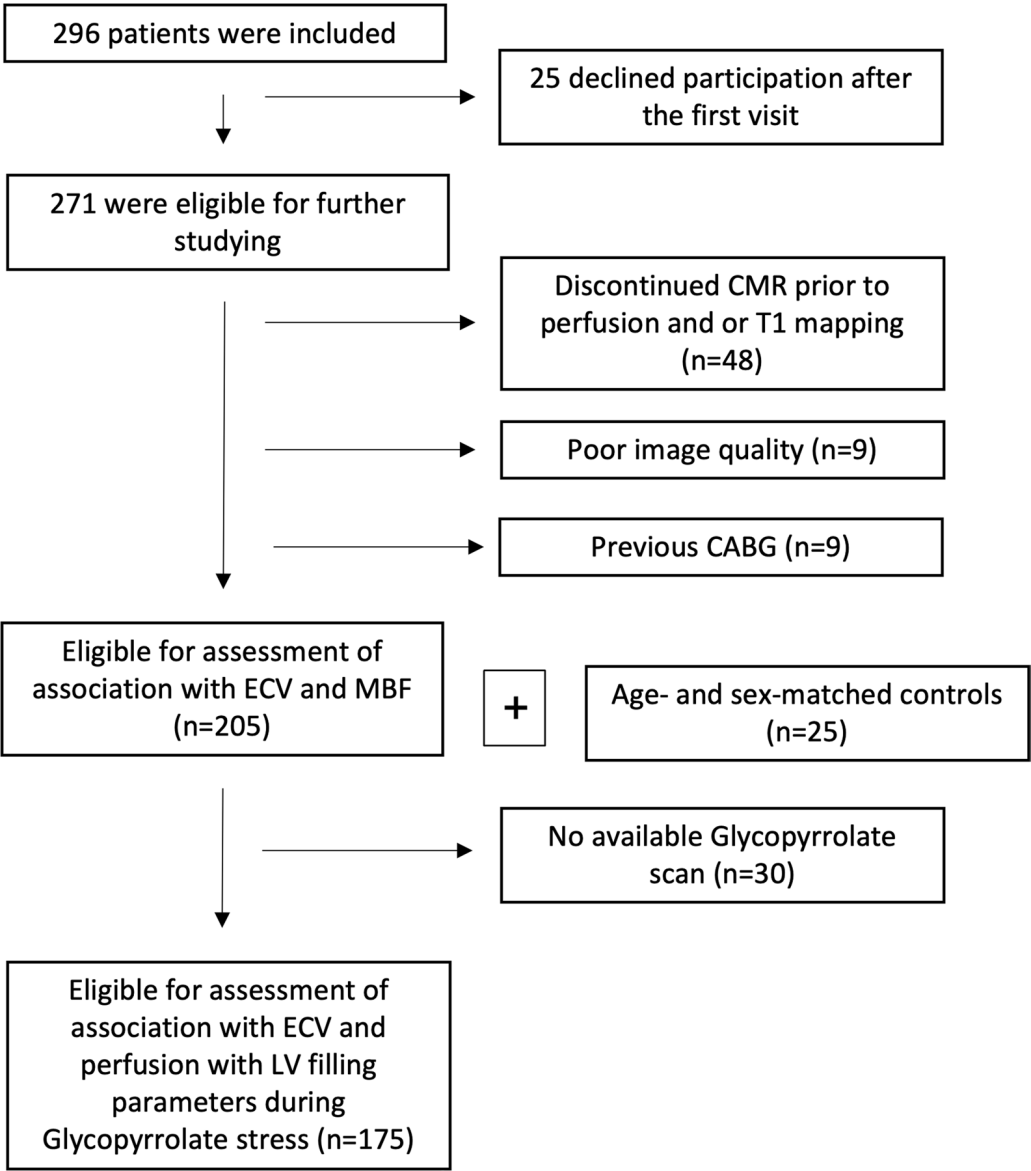
Flow diagram of the patient inclusion

Among 296 patients, 205 had fully analyzable data on ECV, MBF, and MPR. Of these, 175 patients also had analyzable glycopyrrolate chronotropic stress scans. Patient selection is presented in the flow diagram in Fig. 3. A sensitivity analysis found minor differences between patients with an available scan and those without (Additional file 1: Tables S1 and Additional file 2: Table S2).

Table 1 summarizes the patients’ characteristics. The patients with type 2 diabetes were middle-aged (median 60, IQR 52–68), 72% were males, and they had a median diabetes duration of 12 years, IQR 6–18. As compared to control subjects, patients with type 2 diabetes had higher ECV with a larger standard variation indicating a larger spread (29.0 ± 3.28 vs controls; 27.4 ± 2.05%). Estimates for MBF and MPR have been reported in a previous study of 193 of our patients [12], and our results were alike. Patients with type 2 diabetes compared to control subjects had lower ePFR/EDV, LAEF, LA_PEF_, and lateral e´, as well as increased radial peak diastolic strain rates. However, LA maximum volume/BSA was actually decreased, and no differences were found in circumferential and longitudinal peak diastolic strain rates or for average E/e´. 99.5% (204 of the 205) of the patients with type 2 diabetes and all the control subjects were Caucasian.

**Table 1:** Characteristics of the study population

	Controls, n = 25	Patients with type 2 diabetes, n = 205	p
Age, years	57 IQR 50, 64	60 IQR 52, 68	0.2
Sex, male %	17 (68)	148(72)	0.6
Duration of diabetes mellitus, years	–	12 IQR 6, 18	–
Resting heart rate, bpm	59 ± 10	72 ± 11	**
Rate pressure product, beats*mmHg/min	7861 ± 1684	9768 ± 1917	**
BMI, (kg/m^2^)	25 ± 3	31 ± 5	**
HbA1c, (mmol/mol)	35 IQR 33, 37	60 IQR 53, 69	**
eGFR, mL/min/1.73m^2^	87 IQR 80, 90	90 IQR 78, 90	0.7
Hypertension, (%)	4(16)	145(71)	**
Ischemic heart disease, (%)	0	35(17)	0.05
Albuminuria, (%)	–	77(38)	–
Retinopathy, (%)	–	27 (27)	–
Autonomic nephropathy, (%)	–	70 (34)	–
Peripheral neuropathy, (%)	–	84(44)	–
ECV, %	27.4 ± 2.05	29.0 ± 3.28	*
Rest MBF, mL/min/g	0.63 ± 0.11	0.82 ± 0.19	**
Stress MBF, mL/min/g	3.11 ± 0.81	2.44 ± 0.92	*
MPR	5.1 ± 1.5	3.0 ± 1.1	**
ePFR/EDV rest, 1/sec	2.9 ± 0.6	2.4 ± 0.7	*
ePFR/EDV Glycopyrrolate, 1/sec	2.6 IQR 2.0, 3.0	2.2 IQR 1.7, 2.7	*
LA max. volume rest/ BSA mL/m^2^	50 IQR 45, 56	43 IQR 38, 51	*
LAEF rest, %	60 IQR 53, 63	53 IQR 47, 59	*
LA_PEF_ rest, %	30 ± 9	22 ± 9	**
LA max. volume glycopyrrolate/BSA, mL/m^2^	53 IQR 43, 57	39 IQR 35, 46	**
LAEF glycopyrrolate, %	54 ± 9	50 ± 8	**
LA_PEF_ glycopyrrolate, %	25 ± 10	15 ± 8	**
PDSR Circumferential, %/sec	0.79 ± 0.16	0.75 ± 0.17	0.2
PDSR Longitudinal, %/sec	0.82 ± 0.14	0.82 ± 0.18	1.0
PDSR Radial, %/sec	− 2.1 IQR − 2.4, − 1.9	− 1.3 IQR − 1.6, − 1.0	*
Lateral e* (Echo), cm/s	9.6 ± 2.5	8.1 ± 2.0	*
Average E/e* (Echo)	8.3 IQR 7.1, 11.0	8.9 IQR 7.5, 11.3	0.2

BMI, Body mass index; eGFR, estimated glomerular filtration rate; EDV, end-diastolic volume; ECV, extra cellular volume; MBF, myocardial blood flow; MPR, myocardial perfusion reserve; ePFR, early peak filling rate; BSA, body surface area; LA_PEF_, left atrial passive emptying fraction; LAEF, left atrial emptying fraction; PDSR, peak diastolic strain rate; E, Early mitral inflow; Lat e’, lateral myocardial tissue velocity; E/A, ratio between early and late mitral inflow

^*^P < 0.05, **p value < 0.001

The basic and the large multivariable regression model in patients with type 2 diabetes are shown in Table 2. In the basic regression model, both ECV, MBF at rest, MBF at stress, and MPR were associated with LV diastolic but they were associated with different LV diastolic parameters. A 10% increase in ECV was associated with a decrease in ePFR/EDV and an increase in LA_max_/BSA both at rest and stress, as well as a decrease in circumferential and radial peak diastolic strain rates. However, ECV was not associated with Lateral e*, E/e*, or longitudinal peak diastolic strain rates (data not shown in the table). In the large multivariable regression model, ECV was still independent of MPR and other potential confounders associated with all the same LV diastolic parameters as in the basic model.

**Table 2:** Multivariable regression assessing the association of a 10% increase of ECV, MBF rest, MBF stress, and MPR respectively with LV diastolic parameters in patients with type 2 diabetes

Predictive variable	LV filling outcome	Basic model β (95%CI)	p-value	Large model β (95%CI)	p-value
ECV (10% increase)	ePFR/EDV rest	− 3.9% (− 7.1, − 0.6)	0.02	− 4.0% (− 7.5, − 0.4)	0.03
	ePFR/EDV stress	− 6.4% (− 10.7, − 2.1)	0.005	− 7.9% (− 12.5, − 3.0)	0.002
	LA max./BSA rest	5.2% (2.3, 8.2)	< 0.001	4.8% (1.7, 8.0)	0.002
	LA max./BSA stress	6.0% (2.6, 9.4)	< 0.001	5.8% (2.1, 9.7)	0.002
	Circumferential PDSR	− 4.5% (− 7.1, − 1.9)	0.001	− 4.1% (− 6.9, − 1.2)	0.007
	Radial PDSR	0.06%/sec (0.01, 0.1)	0.02	0.07%/sec (0.01, 0.1)	0.01
MBF rest (10% increase)	ePFR/EDV rest	2.9% (1.2, 4.7)	0.001	0.2% (0.05, 0.4)	0.01
	LA_PEF_ stress	− 0.5 percentages point (− 1.0, − 0.03)	0.04	− 0.5percentages point (− 1.1, − 0.007)	0.047
	Circumferential PDSR	2.3% (0.8, 3.8)	0.003	2.0% (0.5, 3.6)	0.01
	Radial PDSR	− 0.04%/sec (− 0.07, − 0.01)	0.003	− 0.03%/sec (− 0.06, − 0.004)	0.03
MBF Stress (10% increase)	LA max./BSA stress	− 1.2% (− 2.2, − 0.1)	0.03	–	0.09
	Circumferential PDSR	1.1% (0.2, 2.0)	0.02	–	0.4
	Lateral e’	1.2% (0.2, 2.2)	0.02	1.4% (0.01, 2.7)	0.03
	Average E/e’	− 1.8% (− 2.8, − 0.8)	< 0.001	− 1.4% (− 2.7, − 0.1)	0.04
MPR (10% increase)	LA max./BSA stress	− 1.0% (− 2.0, − 0.03)	0.045	–	0.08
	Lateral e’	2.3% (0.5, 4.2)	0.01	2.7% (0.2, 5.3)	0.035
	Average E/e’	− 3.4% (− 5.2, − 1.5)	< 0.001	− 2.8% (− 5.4, − 0.3)	0.03

The Association of ECV, MBF rest, MBF stress and MPR with all of the chosen LV diastolic parameters were performed but here the non-Significant associations are not shown. The basic model was adjusted for age, sex, and rate pressure product. The large model was adjusted for age, sex, rate pressure product, duration of diabetes, body mass index, hypertension, ischemic heart disease, albuminuria, retinopathy, autonomic neuropathy, and periphery neuropathy, in addition to ECV and MBF (rest, stress or MPR respectively). Thus, in the large multivariable model, associations with ECV were adjusted for perfusion indices and vice versa

EDV, end-diastolic volume; ECV, extra cellular volume; MBF, myocardial blood flow; MPR, myocardial perfusion reserve; ePFR, early peak filling rate; BSA, body surface area; LA_PEF_, left atrial passive emptying fraction; PDSR, peak diastolic strain rate; E, Early mitral inflow; Lat e’, lateral myocardial tissue velocity

MBF at rest was in the basic model (Table 2) associated with ePFR/EDV at rest but not during stress. Additionally, association with LA_PEF_ during stress, circumferential, and radial peak diastolic strain rates were found for MBF at rest. All of the associations persisted after multivariable adjusting in the large model which included adjusting for ECV. MBF at rest did not associate with LA maximum volume, lateral e*, E/e*, or longitudinal peak diastolic strain rates (data not shown).

MBF at stress associated in the basic model with LA maximum volume indexed to BSA at stress but not at rest, to circumferential peak diastolic strain rates, lateral e*, and Average E/e*. However, after multivariable adjusting in the large model, only the association with lateral e* and average E/e* was significant. MBF at stress did not associate with ePFR/EDV, LAEF or LA_PEF,_ nor did stress MBF associate with longitudinal or radial peak diastolic strain rates (data not shown).

MPR was associated with lateral e* and average E/e* in both the basic model and the large multivariable-adjusted model. The association with LA maximum size was not consistent. MRP did not associate with ePFR/EDV, LAEF, LA_PEF,_ or any of the peak diastolic strain rate parameters (data not shown).

Figure 2 includes a graphic presentation of the four diastolic phases and how the included diastolic parameters relate to the diabetes phases. In addition, we have illustrated which diastolic parameters that were associated with ECV, MBF at stress, and MBF in the large model.

## Discussion

In this work, we investigated the relationship between the myocardial extracellular volume, the myocardial blood flow at rest and during adenosine stress, and the myocardial perfusion reserve and left ventricular diastolic function. The myocardial extracellular volume (which correlates well with myocardial diffuse fibrosis in diabetic rabbits [8]) is an imaging biomarker of interstitial fibrosis. The myocardial blood flow and myocardial perfusion reserve reflect the microvascular circulation. We found, as hypothesized, that the myocardial extracellular volume, the myocardial blood flow, and the myocardial perfusion reserve were associated with left ventricular diastolic function. They were so independent of each other and other known factors associated with diastolic dysfunction. Thus, most associations persisted throughout multivariable adjustments with only small changes in the point estimates. The myocardial extracellular volume was predominantly associated with markers of left ventricular compliance. Myocardial blood flow at stress and myocardial perfusion reserve were primarily associated with cardiomyocyte relaxation as illustrated in Fig. 2.

It is widely demonstrated that diabetes causes interstitial fibrosis and microvascular rarefaction and microvascular dysfunction [29–31]. A general understanding is that these are the underlying factors causing diastolic dysfunction, which is highly prevalent in patients with type 2 diabetes [32–34] and a precursor of HFpEF. The evidence for the development of myocardial interstitial fibrosis stems primarily from animal studies [6, 35] and smaller human biopsy studies [7]. However, a biopsy of the myocardium is an invasive procedure and not without risk; thus, patients in prior biopsy studies were all with known cardiac disease [7]. Studies like ours, where a non-invasive technique is used to study the impact of interstitial fibrosis on left ventricular function, are sparse, perhaps due to this technique being relatively newly developed. An association between the myocardial microvascular function and LV diastolic function has previously been described [10–12, 15, 36, 37] but not independent of the related myocardial fibrosis. Further, studies including a plethora of different diastolic parameters and hence their relative association with microvascular function have not been reported before.

In a CMR study of 135 Chinese patients with type 2 diabetes, an objective similar to ours was studied. The control subjects had similar ECV as ours, but despite the patients being younger with lower BMI and shorter duration of diabetes, the mean ECV in the patients with type 2 diabetes in this cohort was considerably higher (32.6 ± 4.6%) than in our study. In addition to quantifying ECV, they assessed MBF at rest and LV peak diastolic strain rate [11]. In univariable analysis, they found that ECV was associated with longitudinal and radial peak diastolic strain rate. In contrast to our study, after multivariable adjustment, they found that only the association with a longitudinal peak diastolic strain rate was significant [11]. We found that impairment of MBF during adenosine stress and MPR were associated with impaired lateral e’ and that this drove an association with increased average E/e’. The early diastolic myocardial velocity e’ reflects early LV relaxation and restoring forces of the left ventricle [38]. Early myocyte relaxation is an energy-dependent process, and it would therefore seem logical that this would be affected by the cardiomyocyte blood supply.

Longitudinal peak diastolic strain rate is a measure of the same diastolic phase as e´. We did, however, not find an association between stress MBF or MPR and longitudinal peak diastolic stress as was found with lateral e′ and E/e′. In our study, lateral e′ and E/e′ were measured with echo-Doppler whereas longitudinal peak diastolic strain rate was measured with CMR. CMR has a lower temporal resolution than echocardiography. This could be part of the explanation. Thus, if longitudinal peak diastolic strain rate by echo-Doppler would be associated with microvascular function in our subjects cannot be concluded from our study.

Other studies have also shown myocardial microvascular function to be related to cardiomyocyte relaxation. A case–control study of 66 patients using echocardiography showed that both the MPR and e’ were reduced in patients with type 2 diabetes when compared to controls, but no evaluation of ECV or other biomarkers of myocardial fibrosis was performed, and therefore, the relative impact of fibrosis could not be determined [36]. In another study, a (modest) Spearman’s correlation between MPR and circumferential early diastolic strain rate (CMR-Tagging) was demonstrated in 65 patients (19 with diabetes, 30 with prediabetes, and 16 controls) [10]. In the same CMR study described above involving 135 Chinese patients with type 2 diabetes, rest MBF was associated with both longitudinal and circumferential peak diastolic strain rate [11]. In our study, an association of rest MBF with circumferential peak diastolic strain rates, but not with longitudinal peak diastolic strain rate, was found. Taken together, the hypothesis that the microvascular function independently affects early LV relaxation parameters appears reproducible.

As also previously presented [12], patients with type 2 diabetes have higher rest MBF and higher rate pressure product likely reflecting a higher oxygen requirement of the myocardium. Despite this fact, our study showed that increasing rest MBF was associated with signs of enhanced diastolic function with favorable ePFR, circumferential- and radial peak diastolic strain rate. We speculated that the physiologic ability of the myocardium to increase resting MBF in order to preserve LV diastolic function is a sign this myocardium still has a compensatory ability. If in fact high or low resting MBF is associated with poor clinical outcomes remains to be studied in a prospective study design.

We found ECV to be related primarily to measures of LV compliance, and of note, this included a positive association between increasing ECV and increasing maximal LA volume. This is despite the fact that LA maximum volume is lower in patients with type 2 diabetes compared to control subjects shown in our cohort as well as in other studies for example the UK biobank CMR study [39]. This finding may indicate that increasing LA size is still associated with pathological processes of the heart and may therefore be a poor prognostic sign, just as it is in subjects without diabetes.

## Strengths and limitations

To our knowledge, this is the largest cohort study of patients with type 2 diabetes to present data on ECV, MBF at rest, MBF at stress, and MPR with CMR. We included a diastolic stress test since diastolic dysfunction in some patients reveals itself only during stress [22]. We chose to collect a wide variety of clinically used diastolic parameters enabling us to present data on a plethora of diastolic parameters. Thus, our data allowed for the opportunity to understand the relative importance of the different diastolic parameters. However, our study also has important limitations. Our study was cross-sectional, which precludes us from determining causality. We excluded patients with eGFR < 30 mL/min/1.73 m^2^ and patients with persistent or permanent atrial fibrillation, and our results must be interpreted accordingly. Especially concerning the assessment of glycopyrrolate stress, not all available scans were analyzable because we had to exclude patients due to contraindications, intolerance, or poor image quality. A sensitivity analysis indicates that the patients with available glycopyrrolate scans were somewhat healthier than patients without this test (Additional file 2: Table S2). We could still demonstrate a significant impact of ECV on ePFR/EDV and LA_max_/BSA during glycopyrrolate stress, but we may have missed other associations with diastolic stress parameters. In this study, the gadolinium dose for the post-contrast T1 maps was smaller than currently recommended as mentioned in the methods section. However, ECV has not conclusively been shown to be dependent on gadolinium doses, and we included an age- and sex-matched control group which increases the external validity of the study. The ECV values of the control groups were similar to healthy controls of similar age in other publications [11, 21].

In this work, we used adenosine to determine the myocardial microvascular capacity. In comparison with e.g. dobutamine, adenosine produces vasodilation in the myocardium (but does not affect myocardial stress), whereas dobutamine increases myocardial contractility and (indirectly) stresses myocardial oxygen requirements. The usage of adenosine stress in the assessment of the microvascular function has been validated against invasive assessment [40] and is implemented in guidelines [41, 42]. This ability to increase blood flow during adenosine stress-induced hyperemia has in previous work been shown to be significantly decreased in patients with type 2 diabetes. In control subjects, MPR was in our studies increased by a factor of 5.1 whereas it was only increased by a factor 3.0 in patients with type 2 diabetes [15].

## Conclusions

In patients with type 2 diabetes, increased myocardial ECV, a biomarker of interstitial fibrosis, and microvascular dysfunction are associated independently of each other with different aspects of left ventricular diastolic dysfunction. The ECV is predominantly associated with parameters that relate to left ventricular compliance, and MBF at stress and MPR are predominantly associated with the imaging parameters of cardiomyocyte relaxation. These different underlying pathophysiologic features should be considered when imaging these patients both in the clinic and in clinical trials.

## What is already known on this topic

- In animal studies interstitial fibrosis are related to LV diastolic function**.**
- In patients with type 2 diabetes, a sporadic variety of parameters of LV diastolic function have been associated with myocardial perfusion parameters**.**
- ECV and MBF/MPR can be quantified by CMR during the same examination with modern CMR technique

## What this study adds

- Both ECV and MBF/MPR were independently from each other associated with LV diastolic function
- ECV; a imaging biomarker of diffuse fibrosis, was associated with poor LV compliance – increasingly so this amplified during diastolic stress
- Stress MBF and MPR; measures of the myocardial microvascular function, were associated with myocardial relaxation and restoring forces

## Supplementary Information

**Additional file 1: Table S1.** Clinical characteristics of patients that were excluded vs. included in the analysis of all rest parameters both CMR and Echo-Doppler.

**Additional file 2: Table S2.** Clinical characteristics of patients that were excluded vs. included in the analysis of the glycopyrrolate stress parameters.

## Data Availability

Beginning 3 months and ending 5 years from publication, data will be shared with researchers who provide a methodologically sound proposal and who get all the appropriate approvals. Individual participant data that underlie the results and the study protocol can be shared. Proposals should be directed to the corresponding author.

## Abbreviations

LV: Left ventricle
MBF: Myocardial blood flow
MBF: Myocardial perfusion reserve
ECV: Extracellular volume
CMR: Cardiovascular magnetic resonance imaging
ePFR: Early peak filling rate
PDSR: Peak diastolic strain rate
LA: Left atrium
HFpEF: Heart failure with preserved ejection fraction
SGLT-2i: Sodium-glucose cotransporter 2 inhibitors
eGFR: Estimated glomerular filtration rate
RPP: Rate pressure product
LA_max_: Left atrium maximal volume
LA_min_: Left atrium minimal volume
LA_mdv_: Left atrium mid-diastolic volume
