# Supplementary material for: The independent association of myocardial extracellular volume and myocardial blood flow with cardiac diastolic function in patients with type 2 diabetes: a prospective cross-sectional cohort study
Source: Cardiovasc Diabetol. 2023 Mar 31;22:78. doi: 10.1186/s12933-023-01804-9 (PMC10067250; doi:10.1186/s12933-023-01804-9)
Supplement: Supplementary file 1 — Additional file 1: Table S1. Clinical characteristics of patients that were excluded vs. included in the analysis of all rest parameters both CMR and Echo-Doppler. [file 12933_2023_1804_MOESM1_ESM.pdf]

Table S1 Clinical characteristics of patients that were excluded vs. included in the analysis of all rest parameters both CMR and Echo-Doppler

	Diabetes patients who did not have analysable perfusion and T1 (ECV) images<br>N=66	Diabetes population used in this study<br>N=205	p
Age, years	63 IQR 52, 67	60 IQR 52, 68	0.5
Sex, male %	44(67)	148(72)	0.3
Duration of diabetes mellitus, years	11 IQR 6, 17	12 IQR 6, 18	0.4
Systolic blood pressure, mmHg	136 IQR 128, 144	135 IQR 127, 146	0.6
Diastolic blood pressure, mmHg	82 IQR 75, 86	81 IQR 75, 86	0.6
Resting heart rate, bpm	76±13	72±11	0.02
BMI, (kg/m$^{2)}$	31±5	31±5	0.8
HbA1c, (mmol/mol)	63 IQR 54, 73	60 IQR 53, 69	0.3
eGFR, mL/min/1.73m^2	90 IQR 76, 90	90 IQR 78, 90	1.0
New York heart association class, %	I 46(71), II 17(26), III 2(3)	I 164(80), II 33(16), III (7(3)	0.2
Smoker, current or former(%)	50 (76)	136 (67)	0.2
Hypertension, (%)	45 (69)	145 (71)	0.9
Ischemic heart disease, (%)	13 (20)	35 (17)	0.7
Hypercholesterolemia, (%)	39 (60)	129 (63)	0.7
Microalbuminuria, (%)<br>Macroalbuminuria, (%)	23 (37)<br>2 (3)	65 (32)<br>12 (6)	0.6
Simplex retinopathy, (%)<br>Severe retinopathy, (%)	9 (14)<br>9 (14)	27 (14)<br>28 (14)	1.0
Impaired Autonomic nephropathy, (%)<br>Abolished Autonomic nephropathy, (%)	5 (8)<br>22 (35)	16 (8)<br>54 (27)	0.4
Peripheral neuropathy, (%)	26 (40)	84 (44)	0.7
ECV, %			
Rest MBF, mL/min/g	0.82±0.22	0.82±0.19	0.9
Stress MBF, mL/min/g	1.83±0.5	2.44±0.9	0.1
MPR	2.2±0.3	3.0±1.1	0.1
ePFR rest, mL/sec	364±135	366±137	0.9
ePFR Glycopyrrolat, mL/sec	296±101	282±124	0.5
ePFR/LV EDV rest, 1/sec	2.28 IQR 1.97, 2.89	2.37 IQR 1.93, 2.86	1.0
ePFR/LV EDV Glycopyrrolate, 1/sec	2.41 IQR 1.79, 2.92	2.17 IQR 1.72, 2.67	0.1
LA max. volume rest/BSA, mL/m^2	43 IQR 38, 49	43 IQR 38, 51	0.4
LAEF rest, %	53±10	53±9	0.9
LA_{PEF} rest, %	19 IQR 15, 27	21 IQR 15, 28	0.4
LA max. volume glycopyrrolate, mL	39 IQR 35, 45	40 IQR 35, 46	0.6
LAEF glycopyrrolate, %	53±8	50±8	0.03
LA_{PEF} glycopyrrolate, %	14 IQR 10, 21	14 IQR 9, 20	0.6
PDSR Circumferential, %/sec	0.78 IQR 0.65, 0.96	0.75 IQR 0.63, 0.84	0.07
PDSR Longitudinal, %/sec	0.82 IQR 0.70, 0.92	0.82 IQR 0.71, 0.95	0.9
PDSR Radial, %/sec	-1.35±0.42	-1.29±0.43	0.4
Lateral e* (Echo), cm/s	7.9±2.0	8.1±2.0	0.5
Average E/e* (Echo)	9.2 IQR 8.0, 11.1	8.9 IQR 7.5, 11.3	0.4
E/A ratio (Echo)	0.86 IQR 0.73, 1.18	0.90 IQR 0.77, 1.09	0.8
