# Supplementary material for: The independent association of myocardial extracellular volume and myocardial blood flow with cardiac diastolic function in patients with type 2 diabetes: a prospective cross-sectional cohort study
Source: Cardiovasc Diabetol. 2023 Mar 31;22:78. doi: 10.1186/s12933-023-01804-9 (PMC10067250; doi:10.1186/s12933-023-01804-9)
Supplement: Supplementary file 2 — Additional file 2: Table S2. Clinical characteristics of patients that were excluded vs. included in the analysis of the glycopyrrolate stress parameters. [file 12933_2023_1804_MOESM2_ESM.pdf]

Table S2 Clinical characteristics of patients that were excluded vs. included in the analysis of the glycopyrrolate stress parameters

	Diabetes patients who did not have analysable perfusion and T1 (ECV) images and Glycopyrrolate images<br>N=96	Diabetes population used in this part of the study<br>N=175	p
Age, years	63 IQR 56, 70	59 IQR 51, 66	0.006
Sex, male %	70 (73)	122 (70)	0.7
Duration of diabetes mellitus, years	12 IQR 6, 20	12 IQR 6, 18	0.4
Systolic blood pressure, mmHg	134 IQR 127, 141	136 IQR 127, 147	0.3
Diastolic blood pressure, mmHg	80 IQR 74, 86	81 IQR 76, 87	0.08
Resting heart rate, bpm	74±13	73±10	0.2
BMI, (kg/m$^{2)}$	31 IQR 28, 33	31 IQR 28, 34	1.0
HbA1c, (mmol/mol)	61 IQR 54, 70	60 IQR 53, 70	0.6
eGFR, mL/min/1.73m^2	88 IQR 74, 90	90 IQR 80, 90	0.09
New York heart association class, %	I 66(69) II 27 (28) III 2 (2)	I 144 (83) II 23 (13) III 7 (4)	0.008
Smoker, current or former(%)	76 (79)	110 (63)	0.01
Hypertension, (%)	65 (68)	125 (72)	0.7
Ischemic heart disease, (%)	20 (21)	28 (16)	0.4
Hypercholesterolemia, (%)	61 (64)	107 (62	0.8
Microalbuminuria, (%)<br>Macroalbuminuria, (%)	32 (35)<br>5 (5)	56 (32)<br>9 (5)	0.9
Simplex retinopathy, (%)<br>Severe retinopathy, (%)	10 (11)<br>15 (16)	26 (15)<br>22 (13)	0.5
Impaired Autonomic nephropathy, (%)<br>Abolished Autonomic nephropathy, (%)	10 (11)<br>32 (35)	11 (6)<br>44 (25)	0.06
Peripheral neuropathy, (%)	36 (39)	74 (45)	0.4
ECV, %	29±3.8	29±3.1	0.6
Rest MBF, mL/min/g	0.77±0.19	0.83±0.19	0.1
Stress MBF, mL/min/g	2.13±0.90	2.48±0.91	0.04
MPR	2.77±0.89	3.06±1.15	0.9
LGE, Ischemic, (%), non-ischemic (%), both(%)	11(12), 7(8),	21(12), 18 (10)	0.8
ePFR rest, mL/sec	347±128	376±141	0.09
ePFR Glycopyrrolat, mL/sec	296±101	282±124	0.5
ePFR/LV EDV rest, 1/sec	2.17 IQR 1.87, 2.74	2.42 IQR 1.98, 2.92	0.04
ePFR/LV EDV Glycopyrrolate, 1/sec	2.41 IQR 1.79, 2.92	2.17 IQR 1.72, 2.67	0.1
LA max. volume rest/BSA, mL/m^2	43 IQR 39, 50	43 IQR 38, 51	0.9
LAEF rest, %	52±10	53±9	0.2
LA_{PEF} rest, %	19 IQR 14, 26	22 IQR 16, 29	0.02
LA max. volume glycopyrrolate, mL	39 IQR 35, 45	40 IQR 36, 46	0.4
LAEF glycopyrrolate, %	52±8	50±8	0.04
LA_{PEF} glycopyrrolate, %	14 IQR 10, 21	14 IQR 9, 20	0.6
PDSR Circumferential, %/sec	0.77 IQR 0.63, 0.94	0.76 IQR 0.63, 0.84	0.3
PDSR Longitudinal, %/sec	0.80 IQR 0.69, 0.90	0.83 IQR 0.73, 0.95	0.2
PDSR Radial, %/sec	-1.3±0.43	-1.3±0.43	0.9
E (Echo), m/s	0.66 IQR 0.58, 0.78	0.71 IQR 0.59, 0.80	0.2
lateral e* (Echo), cm/s	7.83±1.98	8.15±2.02	0.2
Average E/e* (Echo)	9.17 IQR 7.70, 10.67	8.90 IQR 7.50, 11.38	0.9
E/A ratio (Echo)	0.85 IQR 0.72, 1.12	0.91 IQR 0.77, 1.10	0.2
